# A prognosis model for clear cell renal cell carcinoma based on four necroptosis-related genes

**DOI:** 10.3389/fmed.2022.942991

**Published:** 2022-08-09

**Authors:** Qiangmin Qiu, Yanze Li, Ye Zhang, Yanguang Hou, Juncheng Hu, Lei Wang, Zhiyuan Chen, Yourong Lei, Yang Du, Xiuheng Liu

**Affiliations:** ^1^Department of Urology, Renmin Hospital of Wuhan University, Wuhan, China; ^2^Wuhan University Institute of Urologic Disease, Renmin Hospital of Wuhan University, Wuhan, China; ^3^Department of Infection Prevention and Control, Renmin Hospital of Wuhan University, Wuhan, China

**Keywords:** necroptosis, clear cell renal cell carcinoma, prognosis, platinum drug resistance, genes

## Abstract

Necroptosis is a type of caspase-independent cell death, and it plays a critical role in regulating the development of cancer. To date, little is known about the role of necroptosis-related genes (NRGs) in clear cell renal cell carcinoma (ccRCC). In this study, we downloaded data regarding the expression of NRGs and overall survival (OS) from The Cancer Genome Atlas (TCGA) database and constructed a risk model to determine the prognostic features of necroptosis using COX regression analysis. Patients with ccRCC were divided into low-risk and high-risk groups based on their risk scores. Thereafter, Kaplan–Meier curves were used to evaluate OS, and receiver operating characteristic (ROC) curves were used to determine the accuracy of prediction. Stratified analyses were performed according to different clinical variables. Furthermore, we assessed the correlation between clinical variables and risk scores; the NRGs with differential expression were mainly enriched in positive regulation of intracellular transport and platinum resistance pathways. We constructed prognostic signatures for OS based on four NRGs and showed that the survival time was significantly longer in the low-risk groups than in the high-risk groups (*p* < 0.001). The area of the ROC curve for OS was 0.717, indicating excellent predictive accuracy of the established model. Therefore, a predictive model based on NRGs was constructed, which can predict the prognosis of patients and provides insights into the biological mechanisms underlying necroptosis in patients with ccRCC.

## Introduction

Renal cell carcinoma (RCC) is a common malignancy in humans, and clear cell renal cell carcinoma (ccRCC) is the most dominant type of RCC (1, 2). Surgery is an effective treatment for localized RCC, but limited surgical treatment is available for advanced RCC or metastatic RCC (3, 4). Because of the heterogeneity of tumors, TNM staging is commonly used in clinical practice; however, this is not an optimal technique to predict the prognosis of patients (5). Thus, determining new diagnostic and prognostic markers for ccRCC is urgently required.

Necroptosis is a form of caspase-independent programmed cell death, which recruits downstream mixed-lineage kinase domain-like pseudokinase (MLKL) phosphorylation and localization to the cell membrane through the complex formed by receptor-interacting serine/threonine-protein kinase 3 (RIP3) and receptor-interacting serine/threonine-protein kinase 1 (RIP1), resulting in cell membrane rupture and cell death (6). In addition, necroptosis is involved in tumor recurrence after radiotherapy through the RIP3/RIP1/MLKL/Janus kinase (JNK)/interleukin 8 (IL-8) pathway (7–9).

The role and mechanism of necroptosis in cancer have not been established thus far (10). Cancer cells may survive by inhibiting the expression of key necroptosis genes. The expression of key necroptosis-related genes (NRGs) is often increased in some tumors (11, 12). To date, the studies investigating the role of necroptosis in ccRCC have focused on developing treatment by targeting necroptosis (13, 14), and few studies have reported the function of necroptosis in predicting the prognosis of patients with ccRCC.

In this study, we obtained the expression data of 21 differentially expressed NRGs and the clinical information of patients with ccRCC from The Cancer Genome Atlas (TCGA) database and analyzed the biological functions of these genes. We established an overall survival (OS) model based on the 4 prognostic NRGs and discovered necroptosis-related signatures that could predict the prognosis of patients. Thus, our results showed that these necroptosis-related features are reliable markers of the prognosis in patients with ccRCC.

## Materials and methods

### Data sources

We identified 8 NRGs from the necroptosis gene set M24779.gmt downloaded from the Gene Set Enrichment Analysis (GSEA)^1^ database. Additionally, based on the results of previous studies on necroptosis, we identified 67 NRGs (Supplementary Table 1). We downloaded the RNA sequencing (RNA-seq) data and clinical information of 539 patients with ccRCC from the TCGA database.^2^

### Enrichment analysis

Differentially expressed NRGs were identified using the limma package in R with thresholds of | log2FC| values of > 1 and adjusted *p*-values of < 0.05. To better understand the role of differentially expressed NRGs, the “cluster profiler” package was used for Gene Ontology (GO) and Kyoto Encyclopedia of Genes and Genomes (KEGG) analyses, and the “ggplot2” and “GOplot” packages were used for visualization.

### Construction of a prediction signature

Univariate Cox regression analysis was performed to screen for NRGs in patients with ccRCC, and multivariate COX regression analysis was performed to build a necroptosis-related gene signature using the “survival” R package. The risk score of each patient was calculated, and Kaplan–Meier curves were plotted for survival analysis. The predictive value of the prognostic prediction model was calculated using the area under the curve (AUC) of the receiver operating characteristic (ROC) curve using the “survivalROC” R package. Principal component analysis (PCA) and *t*-distributed stochastic neighbor embedding (t-SNE) were performed using the “stats” R package.

### Experimental validation

To confirm the differences in the expression of the NRGs between ccRCC and normal tissues, we performed experimental validation using specimens obtained from 5 patients with ccRCC who underwent radical nephrectomy or partial nephrectomy from June 2021 to December 2022 at Wuhan University Renmin Hospital. Our study was approved by the internal review board of Renmin Hospital of Wuhan University.

The non-tumor tissues and tumor tissues were fixed in 4% paraformaldehyde, embedded in paraffin, and cut in 4-μm-thick sections. The sections were incubated overnight at 4^°^C with primary antibodies for MYCN (1:150, Abcam, Inc., ab227822), tumor necrosis factor receptor-associated factor 2 (TRAF2) (1:150, Abcam, Inc., ab167163), Bcl-2/adenovirus E1B 19 kDa interacting protein 3 (BNIP3) (1:100, Abcam, Inc., ab205606), and polo-like kinase 1 (PLK1) (1:500, Abcam, Inc., ab155095), and with secondary antibody for 30 min at 37^°^C. The sections were incubated with 3,3′-Diaminobenzidine (DAB) chromogen for 8 min and followed by hematoxylin and bluing reagent counterstaining. Under a microscope, five different fields of view were randomly selected to assess staining intensity. Two pathologists independently assessed the slides of immunohistochemical staining.

### Statistical analysis

All statistical analyses were performed using the R language (version 4.1.2). The rank correlation among the different variables was further assessed using the Pearson correlation coefficient. In addition, independent *t*-tests were performed to compare gene expressions among different tissues. All statistical *p*-values were two-tailed, with *p*-values of < 0.05 indicating statistical significance.

## Results

### Identification of distinct necroptosis-related genes in non-tumor and clear cell renal cell carcinoma groups

**FIGURE 1:**
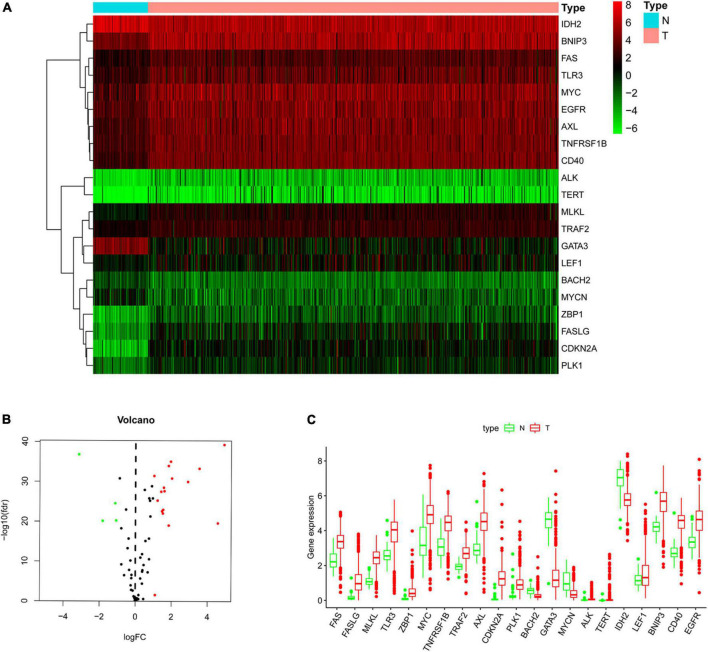
Differentially expressed NRGs in non-tumor and clear cell renal cell carcinoma (ccRCC) tissues. **(A)** Heatmap demonstrating 21 differentially expressed genes; **(B)** volcano map demonstrating 21 differentially expressed genes; **(C)** box plot of genes in non-tumor and ccRCC tissues. Green and red show non-tumor and tumor tissues, respectively.

The flow chart of our research process is shown in Supplementary Figure 1. The RNA-seq data and clinical information of 539 patients were downloaded from the TCGA database, and the RNA expression data of 67 NRGs were examined. A total of 21 NRGs were identified with significant differential expression between the non-tumor and ccRCC groups, including 4 downregulated (*MYNC*, *GATA3*, *IDH2*, and *BACH2*) and 17 upregulated (*TRAF2*, *LEF1*, *FAS*, *TNFRSF1B*, *EGFR*, *MYC*, *TLR3*, *BNIP3*, *AXL*, *CD40*, *ALK*, *MLKL*, *PLK1*, *ZBP1*, *FASLG*, *TERT*, and *CDKN2A*) genes (Figure 1A). The screening criteria were set to | log2 (FC)| > 1 and FDR < 0.05. Box and volcano plots were created to compare the expression of the differentially expressed NRGs between non-tumor and ccRCC tissues (Figures 1B,C).

### Enrichment analysis

**FIGURE 2:**
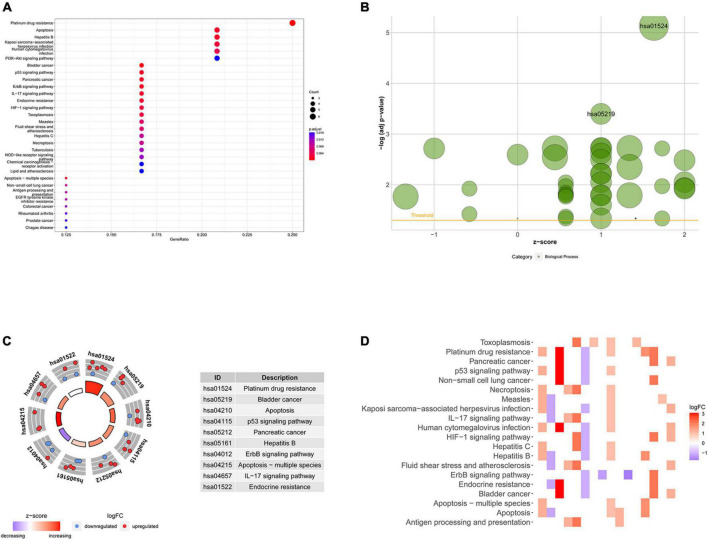
Kyoto Encyclopedia of Genes and Genomes (KEGG) analysis of differentially expressed necroptosis-related genes (NRGs). **(A)** Bubble chart of the top 30 enrichment pathways; **(B)** bubble diagram of enriched KEGG pathways; **(C)** circle diagram of enriched KEGG pathways; **(D)** heatmap of enrichment analysis. The color of each block represents the | log2 (FC)| values.

**FIGURE 3:**
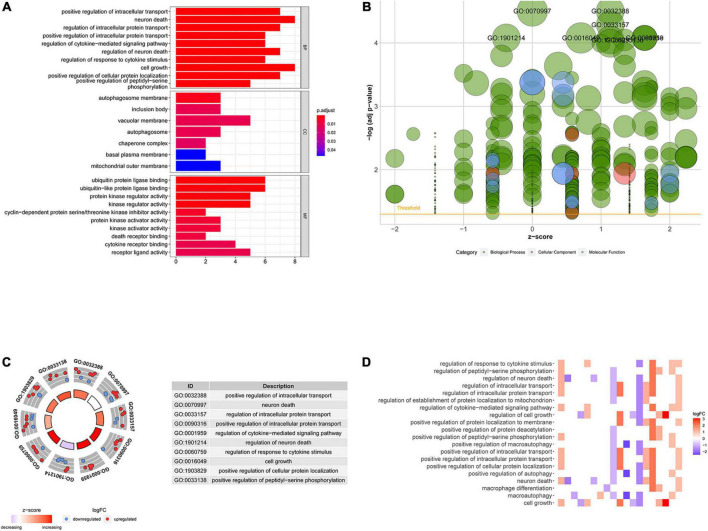
Gene ontology (GO) analysis of differentially expressed NRGs. **(A)** Histogram demonstrating the top 10 enrichment pathways in biological process (BP), cellular component (CC), molecular function (MF); **(B)** bubble plot of GO terms; red denotes cellular components, green denotes biological processes and blue denotes molecular functions; **(C)** circle diagram demonstrating the top 10 significant enriched pathways; **(D)** heatmap demonstrating the relationship between NRGs and GO enrichment. The color of each block represents the | log2 (FC)| values.

Based on the differential expression of NRGs, KEGG, and GO enrichment analyses were performed. NRGs were found to be associated with the following KEGG pathways: platinum drug resistance, bladder cancer, apoptosis, p53 signaling pathway, pancreatic cancer, hepatitis B, erbB signaling pathway, apoptosis-multiple species, interleukin 17 (IL-17) signaling pathway, and endocrine resistance (Figure 2A). The results are shown in Figures 2B–D. The top 10 terms of biological process (BP), cellular component (CC), and molecular function (MF) are shown in Figure 3A, including positive regulation of intracellular transport, neuronal death, regulation of intracellular protein transport, positive regulation of intracellular protein transport, regulation of cytokine-mediated signaling pathway, regulation of neuronal death, regulation of response to cytokine stimulus, cell growth, positive regulation of cellular protein localization, and positive regulation of peptidyl-serine phosphorylation, implying that these prognosis-related genes are associated with necroptosis. The bubble plot, circle diagram, and heatmap are shown in Figures 3B–D, respectively.

### Identification of prognosis-related necroptosis-related genes

**FIGURE 4:**
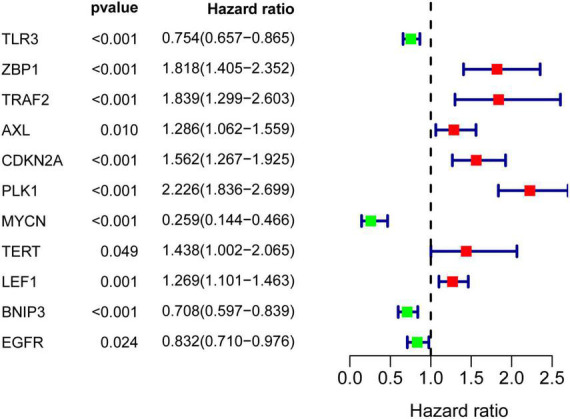
Univariate Cox regression analysis of differentially expressed NRGs.

Results of univariate Cox regression analysis showed that a total of 11 NRGs were significantly associated with OS (Figure 4). Thereafter, 4 NRGs were selected *via* multivariate Cox regression analysis for constructing a prognostic signature for OS (Table 1). OS-related prediction model = (0.2407*TRAF2 expression) + (0.5846*PLK1 expression) + (–0.9912*MYCN expression) + (–0.1837*BNIP3 expression).

**TABLE 1:** Multivariate Cox regression analysis of prognostic necroptosis-related genes (NRGs).

Gene	Coef	HR	HR.95L	HR.95H	*P*-value
*TRAF2*	0.240717	1.272161	0.911441	1.775643	0.157094
*PLK1*	0.584623	1.794315	1.44776	2.223824	9.33E-08
*MYCN*	–0.99115	0.371151	0.206099	0.668382	0.000959
*BNIP3*	–0.18369	0.832195	0.693591	0.998496	0.048137

Coef, coefficient; HR, hazard ratio; CI, confidence interval.

### Construction of prognosis prediction model

**FIGURE 5:**
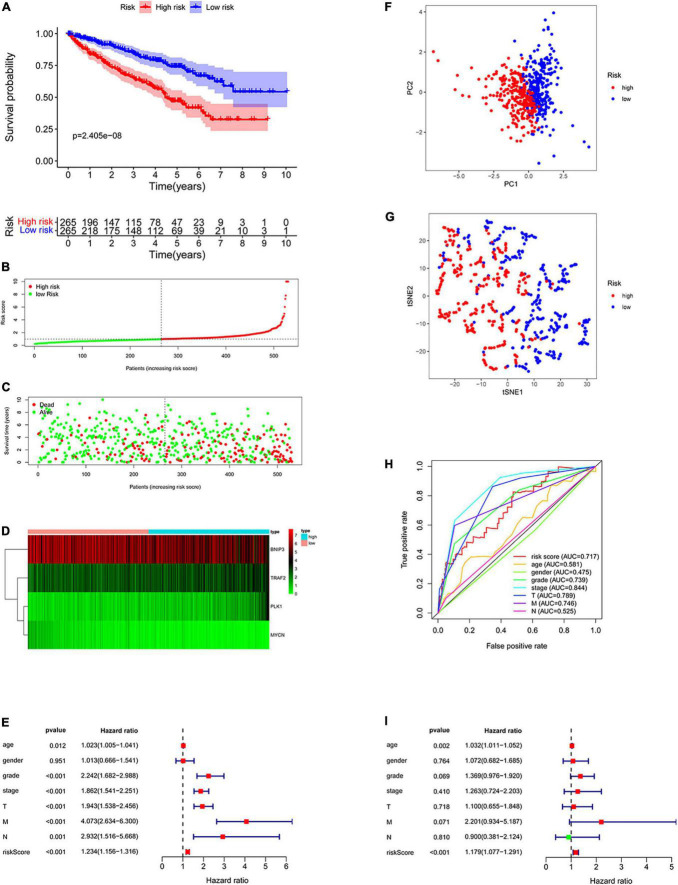
Construction of the prognostic prediction model. **(A)** The Kaplan–Meier survival curve of low-risk groups; **(B)** prognostic model distribution of patients with ccRCC; **(C)** the survival status of patients in the low- and high-risk groups; **(D)** the expression of the four NRGs; **(E)** univariate Cox regression analysis; **(F)** principal component analysis (PCA) plot of the prognosis model; **(G)** *t*-distributed stochastic neighbor embedding (t-SNE) analysis of the prognosis model; **(H)** receiver operating characteristic (ROC) curve showing the predictive precision of the prognosis model; **(I)** multivariate Cox regression analysis.

All patients with ccRCC were divided into high-risk and low-risk groups according to the median risk score (Figure 5B). The scatter diagram shows the survival status of the patients (Figure 5C). The results showed that the patients in low-risk groups had a longer survival time than those in the high-risk groups (*p* = 2.405e-08, Figure 5A). The heatmap showed the expression of the four genes in the high- and low-risk groups (Figure 5D). Furthermore, we used Cox regression analysis to assess the precision of the prognosis model. Except for sex, other factors were significantly correlated with OS in univariate Cox regression analysis (Figures 5E,I). Multivariate Cox regression analysis showed that age and risk score could be used as independent factors and affected the prognosis of ccRCC. Then, we performed the ROC analysis. The AUC was 0.717, demonstrating excellent predictive precision of the model (Figure 5H). PCA and t-SNE analysis revealed a significant distribution difference in the patients with different risk scores (Figures 5F,G).

### Stratified analysis of different subgroups

**FIGURE 6:**
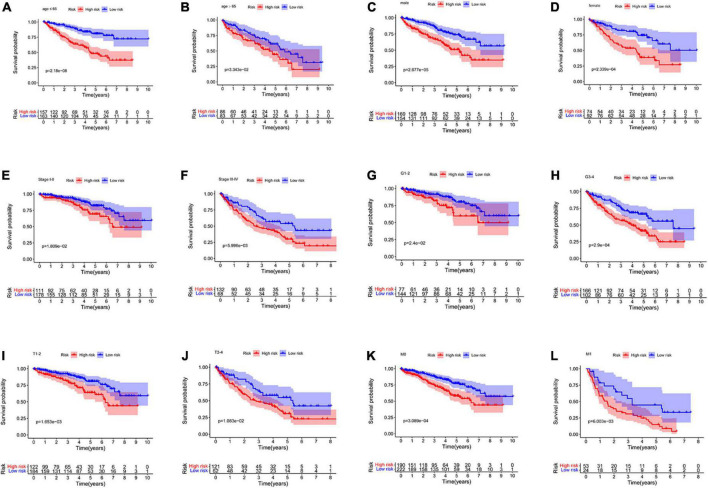
Kaplan–Meier curves for the high- and low-risk groups in different subgroups. **(A)** age ≤ 65 years; **(B)** age > 65 years; **(C)** men; **(D)** women; **(E)** stage I–II; **(F)** stage III–IV; **(G)** grade 1–2; **(H)** grade 3–4; **(I)** T 1–2; **(J)** T 3–4; **(K)** M0; **(L)** M1. M, metastasis; T, tumor size.

The prognosis prediction model could predict OS in different subgroups divided based on age (≤ 65 years and > 65 years), sex (men and women), tumor grade (Grade 1–2 and Grade 3–4), M stage (M0 and M1), tumor stage (Stage I–II and Stage III–IV), and T stage (T 1–2 and T 3–4 stage) (all *p* < 0.05, Figure 6). The model constructed using the four NRGs could predict the prognosis of patients with ccRCC without using clinicopathological factors.

### Clinical correlation analysis

**FIGURE 7:**
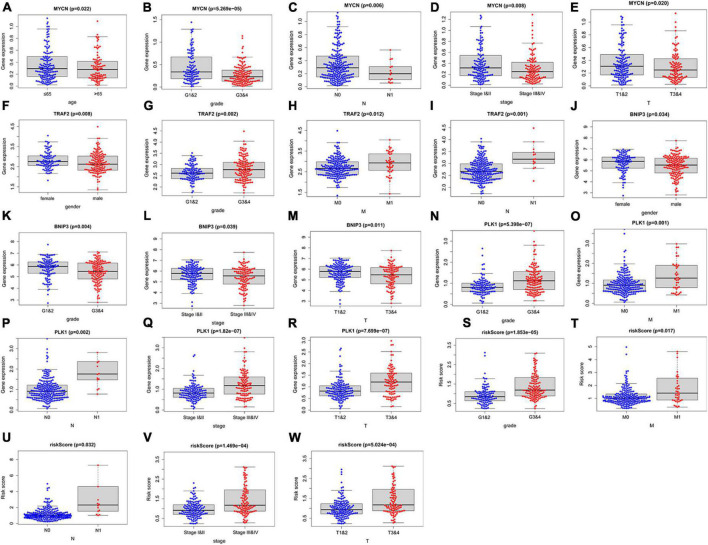
Correlation between the 4 NRGs, necroptosis-related prognostic signature and clinical factors. The expression of *MYCN* in different groups with different age **(A)** grade **(B)**, *N* **(C)**, stage **(D)**, T **(E)**; the expression of *TRAF2* in different groups with distinct sex **(F)**, grade **(G)**, M **(H)**, N **(I)**; the expression of *BINP2* in different groups with different sex **(J)**, grade **(K)**, stage **(L)**, T **(M)**; the expression of *PLK1* and risk-score in different groups with different grade **(N,S)** M **(O,T)**, N **(P,U)**, stage **(Q,V)**, T **(R,W)**.

**FIGURE 8:**
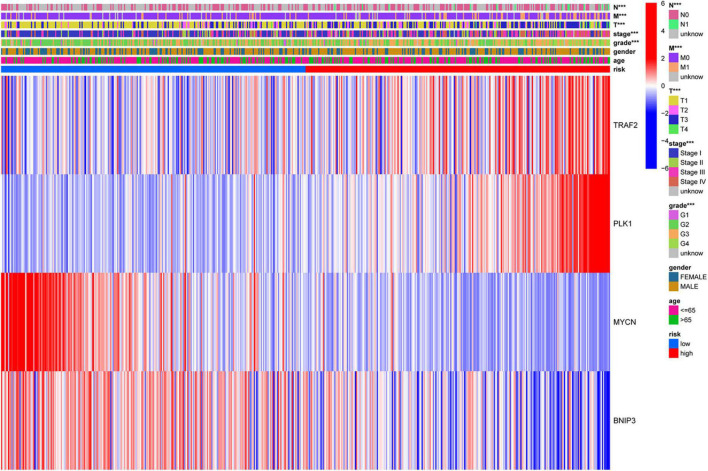
Correlation of risk group and clinicopathological characteristics. ***p < 0.001.

To investigate the clinical value of the necroptosis-related prognostic signature in ccRCC, we analyzed the correlations among the 4 NRGs, necroptosis-related prognostic signature, and clinical factors. The results demonstrated that *MYCN* was significantly associated with age, grade, stage, N, and T (Figures 7A–E); *TRAF2* was significantly associated with sex, grade, N, and M (Figures 7F–I); *BNIP3* was significantly associated with sex, grade, stage, and T (Figures 7J–M). In addition, risk score and *PLK1* were both significantly associated with grade, stage, T, N, and M (Figures 7N–W). The correlations among the 4 NRGs, necroptosis-related prognostic signatures, and clinical factors are shown in Figure 8.

### Validation of the predictive value and expression of necroptosis-related genes

**FIGURE 9:**
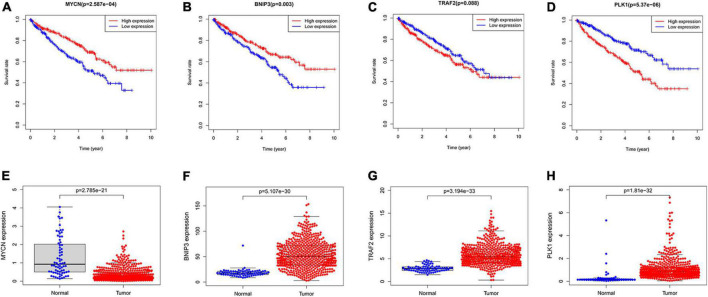
The predictive value of the four NRGs in ccRCC. **(A–D)** Kaplan–Meier survival curve analysis verified the prognostic value of the four NRGs in ccRCC; **(E–H)** verification of the RNA expression of the 4 genes between non-tumor tissues and ccRCC.

We used Kaplan–Meier survival curve analysis to validate the predictive value of the 4 genes. *BNIP3*, *PLK1*, and *MYCN* had excellent prognostic values (Figures 9A,B,D). *TRAF2* has a *p*-value of 0.088 (Figure 9C). Subsequently, our results showed the difference in the expression of these genes between non-tumor and ccRCC tissues (Figures 9E–H).

### Validation of the expression of the four necroptosis-related genes

**FIGURE 10:**
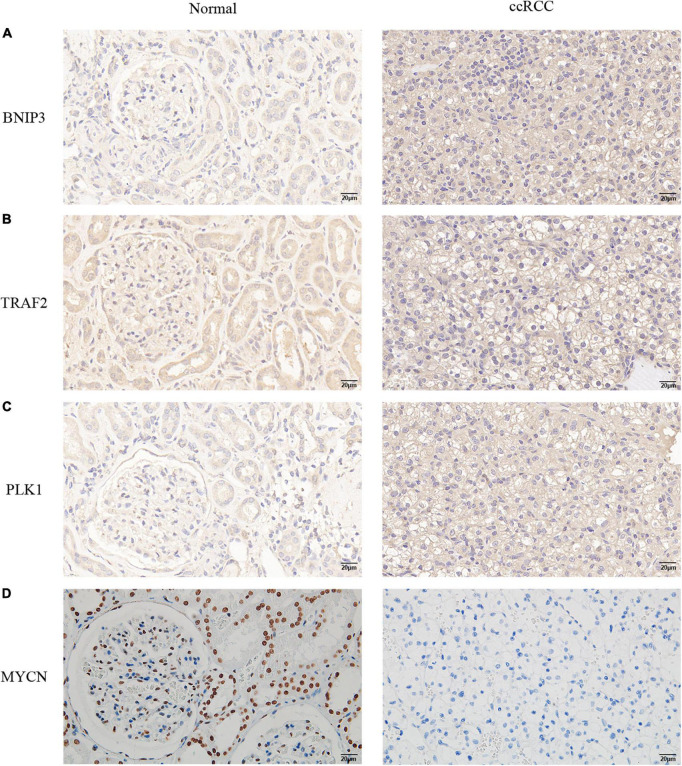
Verification of the protein expression levels of the four NRGs in non-tumor tissues and ccRCC tissues. **(A)** BINP3, **(B)** TRAF2, **(C)** PLK1, **(D)** MYCN.

We performed IHC validation in clinical specimens. The results showed that the expression of BNIP3, TRAF2, and PLK1 proteins was higher in ccRCC tissues (Figures 10A–C). However, the expression of the MYCN protein was low in tumor tissues (Figure 10D). Thus, our results confirmed the accuracy of the established prognostic model.

## Discussion

The ccRCC is prone to recurrence and has a poor prognosis (15). Thus, the development of precise diagnostic and therapeutic biomarkers is urgently required. Necroptosis is a newly discovered form of regulated necrosis. An increasing number of studies have demonstrated that necroptosis plays a critical role in cancer initiation, progression, and metastasis (16, 17). However, the studies performed to date have focused on the role of NRGs in the development of cancer and using them as a therapeutic target (18, 19), and no study has focused on the prognostic value of NRGs in cancer. In this study, we assessed the relationship between NRGs and the prognosis of patients with ccRCC. In addition, we constructed a prognostic model for ccRCC and validated the predictive value of the model.

We screened out 21 differentially expressed genes from 67 NRGs and compared their expression in non-tumor and tumor tissues, including 4 downregulated (*GATA3*, *MYCN*, *IDH2*, and *BACH2*) and 17 upregulated NRGs (*TRAF2*, *LEF1*, *FAS*, *TNFRSF1B*, *EGFR*, *MYC*, *TLR3*, *BNIP3*, *AXL*, *CD40*, *ALK*, *MLKL*, *PLK1*, *ZBP1*, *FASLG*, *TERT*, and *CDKN2A*). Additionally, we performed a functional analysis of the NRGs, and our results indicated that these NRGs were involved in platinum drug resistance, bladder cancer, apoptosis, p53 signaling pathway, pancreatic cancer, hepatitis B, erbB signaling pathway, apoptosis-multiple species, IL-17 signaling pathway, and endocrine resistance. A previous study showed that the sensitivity of ccRCC to cisplatin-induced necroptosis could be regulated by modulating the CAPN4-CNOT3 axis (3). Additionally, several studies have demonstrated that the p53 signaling pathway plays an essential role in the initiation and progression of ccRCC (6). These results suggest that these 21 NRGs may play a significant role in the development of ccRCC.

A total of 11 NRGs were confirmed in univariate Cox regression analysis. Four OS-related NRGs (*MYCN*, *BNIP3*, *TRAF2*, and *PLK1*) were detected by further multivariate Cox regression analysis, and we established OS-related prediction models using the four genes, which could be an independent prognostic factor for the patients with ccRCC. A previous study showed that *PLK1*-mediated *MCM3* phosphorylation regulates proliferation and apoptosis in ccRCC (20). Dufie et al. also showed that *HIF-2* promotes PLK1 expression in ccRCC, which mediates metastasis and resistance to targeted drugs in ccRCC (21). The Myc families of transcription factors (TFs), consisting of *MYC*, *MYCN*, and *MYCL*, are together the most commonly altered oncogenes in cancer (22, 23). Mastropasqua et al. revealed the influence of the N-MYC-miRNAs-TRIM8-p53 axis on the efficacy of cancer treatments in ccRCC (24). One study showed that histone deacetylation is most likely to cause *BNIP3* inactivation in ccRCC, which leads to the suppression of apoptosis and promotes the growth of tumors in ccRCC (25, 26). These findings are consistent with those observed in our study. The relationship between *TRAF2* and ccRCC remains to be clarified and may be related to immunity (27, 28).

In summary, we constructed a prognosis prediction model using the TCGA database and performed validation of the model. We identified a total of four OS-related NRGs (*MYCN*, *BINP3*, *TRAF2*, and *PLK1*). Therefore, these 4 NRGs are capable of acting as promising prognostic and diagnostic biomarkers for patients with ccRCC.

## Data availability statement

The original contributions presented in this study are included in the article/Supplementary material, further inquiries can be directed to the corresponding author/s.

## Author contributions

YD, YRL, and XL conceived, designed the study, and supervised the study. QQ, YZL, and YZ wrote the manuscript, performed the experiments, analyzed, and interpreted the data. YH, JH, LW, and ZC provided technical support. All authors contributed to the article and approved the submitted version.

## Footnotes

1
http://www.gsea-msigdb.org/gsea/index.jsp

2
https://portal.gdc.cancer.gov/
