# Supplementary figures and images for: A prognosis model for clear cell renal cell carcinoma based on four necroptosis-related genes
Source: Front Med (Lausanne). 2022 Aug 9;9:942991. doi: 10.3389/fmed.2022.942991 (PMC9395686; doi:10.3389/fmed.2022.942991)

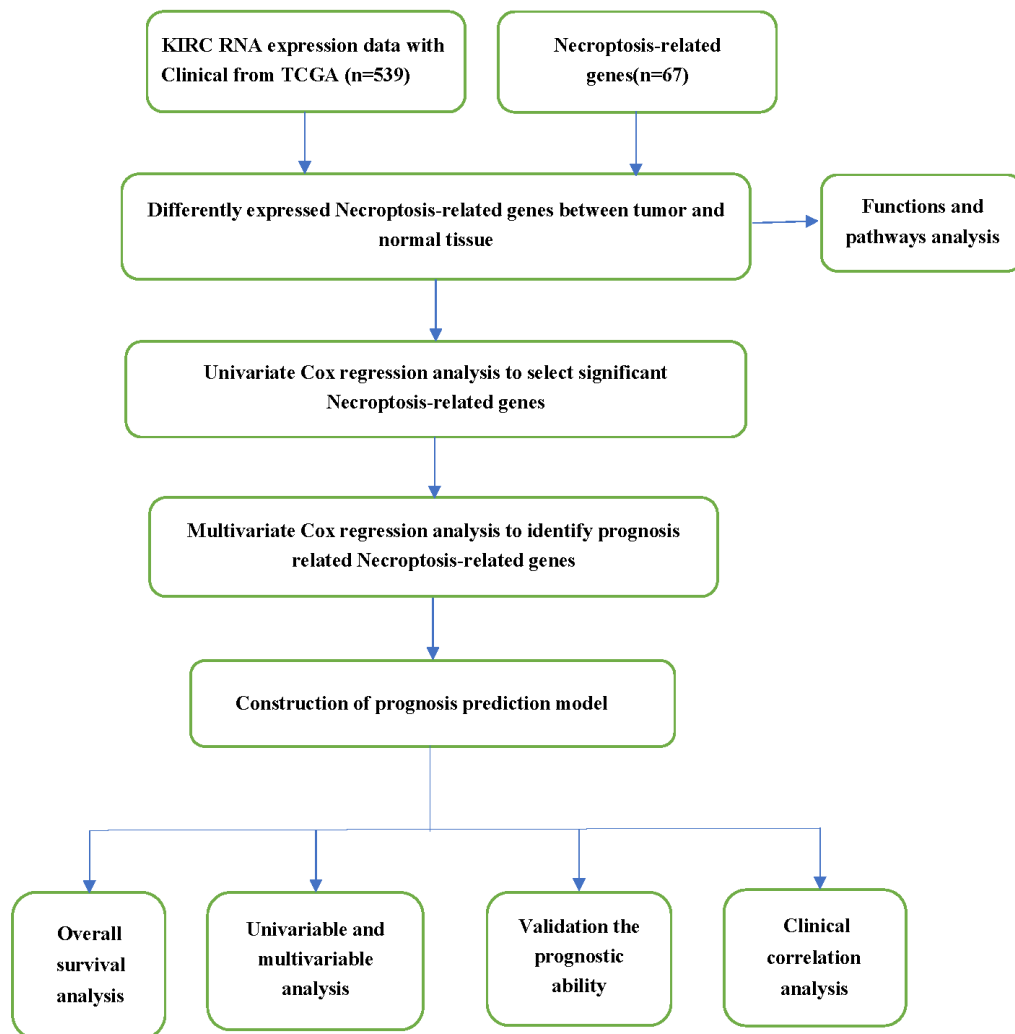

**Figure S1.** The flowchart of the study.

Supplement: Supplementary file 2 [file Image_1.pdf]
